# Performance of a Point-of-Care Test Kit (Anigen Rapid^®^) to Diagnose Feline Immunodeficiency Virus (FIV) Infection in Domestic Cats Using Saliva Instead of Blood in Australia

**DOI:** 10.3390/vetsci12010035

**Published:** 2025-01-09

**Authors:** Jennifer Green, Adele Scannell, Evelyn Hall, Mark E. Westman

**Affiliations:** 1 Sydney School of Veterinary Science, Faculty of Science, The University of Sydney, Sydney, NSW 2006, Australia; evelyn.hall@sydney.edu.au (E.H.); mark.westman@sydney.edu.au (M.E.W.); 2 Animal Aid, Coldstream, VIC 3113, Australia; welfaredirector@animalaid.org.au

**Keywords:** antibodies, Australia, diagnosis, feline immunodeficiency virus, FIV, shelters, vaccination, veterinary science

## Abstract

The primary aim of this study was to determine the accuracy of saliva as a proxy for blood in cats using Anigen Rapid^®^ FIV point-of-care (PoC) kits and as an easy collection technique applicable for all veterinary clinics and shelters. A secondary aim was to report FIV prevalence in various Australian states/territories and key cat risk factors associated with FIV infection. In total, 382 cats were recruited from patients presenting to private, shelter and teaching hospital veterinary clinics in Australia. Information collected for each cat included age, sex, neuter status, postcode, and health status (‘healthy’ versus ‘sick’). The traditional blood testing technique with Anigen Rapid^®^ was used to determine the FIV status of cats. Comparative saliva testing found 48/382 (12.5%) cats were FIV-positive. Test sensitivity for Anigen Rapid^®^ FIV using saliva was 84.2% (48/57; 95% CI 80.6 to 87.9). Test specificity for Anigen Rapid^®^ FIV using saliva was 100% (325/325). The two significant cat risk factors for FIV infection were sex (male) and health status (sick). In terms of demographic information, prevalence in Tasmania and the Northen Territory was reported for the first time. This study proves the ability to utilise cheap and readily available consumables for less invasive FIV testing purposes by using saliva instead of blood, for example, when screening cat before adoption in shelters and in cats prior to annual FIV re-vaccination.

## Simple Summary

Feline immunodeficiency virus (FIV) is an important disease in domestic cats that can have significant impacts on the health and lifestyle of infected individuals. Rapid cage-side testing yielding accurate results is currently possible using blood samples collected from individual cats; however, this can be a stressful and invasive test procedure for cats. The use of saliva as a sample instead of blood would provide a less stressful approach to testing, particularly in shelters where large scale screening in healthy cats for infection is often performed. This study compared the results from traditional blood testing with a practical and simple saliva-based technique using Anigen Rapid^®^ FIV kits. In 382 cats, saliva testing was found to be highly specific (100%) and moderately sensitive (84.2%). A total of 9 cats out of 57 cats tested falsely negative. We believe these results provide good evidence for saliva testing to diagnose FIV infection in some populations of cats, including shelter cats. The saliva testing method may also allow the increased testing of high-risk individuals, particularly male, unwell cats, in both private clinics and shelters.

## 1. Introduction

Feline immunodeficiency virus (FIV) is a common retroviral infection of cats worldwide, clinically stereotypified by the territorial, entire, male cat [1,2]. FIV is transmitted horizontally between cats, most commonly through antagonistic biting, with the large canine teeth inoculating the virus from infected cats into the subcutaneous tissue of uninfected cats [3]. Many scenarios exist whereby a veterinarian might want to know the FIV status of a cat. According to the American Association of Feline Practitioners (AAFP), cats should be tested for FIV as soon as possible after they are acquired, prior to FIV vaccination, after exposure to a FIV-infected cat, and when clinical illness occurs. Animal shelters may also screen for FIV infection prior to adoption, since the greatest risk to FIV spread in a household is in the initial period of introduction of a new cat to a household [1].

While any invasive test of a cat can be challenging, it is generally acknowledged that obtaining a sample of saliva is easier, safer and less stressful for the animal than obtaining a blood sample [4]. Although saliva testing has been utilised for human immunodeficiency virus (HIV-1) testing in humans for decades [5], and surged in popularity during COVID-19, it is a relatively new and growing field in veterinary medicine, including for FIV detection [6].

Whole saliva is made up of a combination of fluid from the salivary glands and crevicular fluid. The saliva itself is from the parotid gland (*Glandula parotis*) and the mandibular gland (*Glandula mandibularis*), as well as some smaller salivary glands. The fluid from these glands is primarily water and includes trace amounts of locally produced immunoglobulins (primarily IgA, but also IgM and IgG) and mucin, proteins, and digestive enzymes [6,7]. Crevicular fluid originates from the capillary network under the buccal mucosa and gingival connective tissue and exudes into the dentogingival space. It has an antibody composition similar to that of plasma and accounts for most of the IgM and IgG found in whole saliva [8]. Given this combination of salivary fluid and crevicular fluid, whole saliva can therefore be considered a transudate of plasma [8].

The earliest studies into the diagnosis of FIV using saliva in the 1990s investigated the presence of FIV viral RNA (vRNA), proviral DNA, and antibodies in saliva samples from experimentally infected cats but were hampered by technological limitations [9,10]. More recently, the use of real-time (q)PCR and microsphere immunoassays have enabled the qualification and quantification of vRNA and anti-FIV IgG in saliva [11,12]. These laboratory-based assays, however, are not helpful for practitioners and shelters wanting rapid patient-side testing [7].

The Anigen Rapid^®^ FIV point-of-care (PoC) test kit (BioNote, Hwaseong-si, Gyeonggi-do, Republic of Korea) accurately detects anti-FIV antibodies in the blood of infected cats in Australia, with researchers reporting 100% sensitivity and 100% specificity using fresh whole blood [13]. Although the PoC test was developed for diagnosis using blood samples (either whole blood, plasma, or serum), the same researchers trialled Anigen Rapid^®^ for the detection of FIV with saliva. Using a centrifugation technique to concentrate saliva samples collected with a dry cotton swab, Anigen Rapid^®^ FIV had 96% sensitivity and 100% specificity [12]. Most veterinary clinics and shelters, however, do not possess the equipment required for this technique.

The primary aim of this study, therefore, was to determine the accuracy of saliva as a proxy for blood in cats of unknown FIV status using Anigen Rapid^®^ FIV PoC kits and a simpler collection technique applicable for all veterinary clinics and shelters. A secondary aim was to report FIV prevalence in various Australian states/territories, including one state (Tasmania) and one territory (Northern Territory) for which this information was not previously available. Additionally, statistical modelling was able to determine key cat risk factors associated with FIV infection in Australia.

## 2. Materials and Methods

### 2.1. Study Population

Cats of unknown FIV status were recruited from patients presenting to 20 private veterinary clinics, a university teaching hospital, and two shelter practices across four states (New South Wales [NSW], Victoria [VIC], South Australia [SA], and Tasmania [TAS]) and one territory (Northern Territory [NT]) in Australia. Cats were tested as part of routine diagnostic testing. Information for each cat was recorded by the attending veterinarian including age; sex; neuter status; postcode; reason for FIV testing; ‘healthy’ or ‘sick’, depending on the clinical presentation or the reason for blood collection; Anigen Rapid^®^ FIV result with blood; and Anigen Rapid^®^ FIV result with saliva. By definition, a ‘sick’ cat was a cat with a health concern deviating from a normal state of wellness. This included cat fight abscesses, dental disease, trauma, hormonal dysfunction, and lethargy.

### 2.2. Blood Collection and Anigen Rapid^®^ FIV PoC Testing Using Blood

Blood was collected by jugular or cephalic venipuncture and transferred to an ethylenediaminetetraacetic acid (EDTA) tube. Fresh EDTA whole blood was then used to determine the FIV status of the cat using an Anigen Rapid^®^ FIV PoC test, according to the manufacturer’s instructions. Anigen Rapid^®^ FIV (BioNote, Gyeonggi-do, Republic of Korea) is a portable lateral flow test kit composed of a nitrocellulose membrane that detects antibodies directed against the FIV transmembrane glycoprotein (gp40) [14]. This study utilised Anigen Rapid^®^ FIV kits (Bionote) that were purchased by Boehringer Ingelheim Animal Health Australia as part of their support of independent feline retroviral research in Australia.

The result from Anigen Rapid^®^ FIV testing was read after 10 min by two testers. If the result window did not show lateral flow of solution after 2 min, an extra drop of buffer solution was added and/or the test kit was tapped a couple of times, as per manufacturer’s instructions.

Any positive FIV result was not confirmed with additional testing and was taken as the cat’s true FIV infection status.

### 2.3. Saliva Collection and Anigen Rapid^®^ FIV PoC Testing Using Saliva

**Figure 1:**
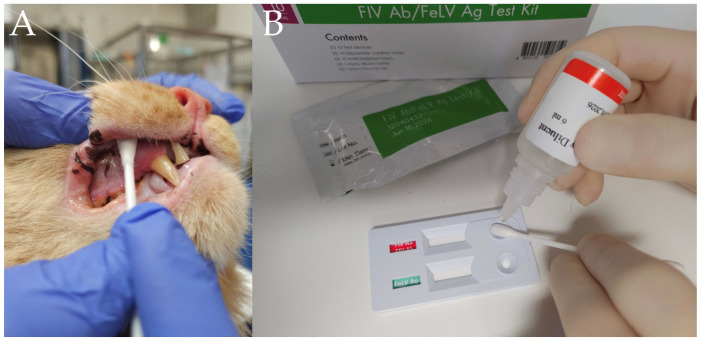
(**A**) Cotton tip placement against the buccal mucosa for saliva collection. (**B**) The cotton tip was positioned directly on the sample well of the Anigen Rapid^®^ FIV kit and test buffer was applied while the cotton swab was gently rotated. The result was read 10 min later by two observers.

A ‘direct’ saliva collection technique, based on a previous pilot study in Australia involving 14 cats, was utilised [12]. Saliva was collected by rubbing a dry and clean cotton tip swab against the buccal mucosa on each side of the mouth, with the cheek pressed gently against the dental arcade while slowly twisting the swab, for approximately 10 s per side (Figure 1A).

For testing with the Anigen Rapid^®^ FIV PoC kit, the saliva-soaked tip of the cotton swab was directly applied to the sample well spot and twice the manufacturer recommended volume of conjugate buffer (i.e., 4 drops instead of 2) slowly added directly onto the saliva-coated swab tip over 10 s. The cotton tip was gently rolled over the sample well spot while the buffer was added (Figure 1B).

The saliva FIV result was read 10 min later by two testers, at least one of whom was blinded to the blood FIV result. Although the manufacturer does not provide guidance for the interpretation of ‘faint positive’ results, any visible band at the ‘T’ (test) line was considered positive.

A standard operating procedure (SOP) video was recorded by the authors and provided to study participants to enable consistent sample collection and FIV testing across the multiple recruitment sites.

### 2.4. Statistical Analysis

Test sensitivity for Anigen Rapid^®^ FIV using saliva was calculated using the following formula: (sensitivity = true-positives/[true-positives + false-negatives] × 100).

Test specificity for Anigen Rapid^®^ FIV using saliva was calculated using the following formula: (specificity = true-negatives/[true-negatives + false-positives] × 100).

Microsoft Excel^®^ (Microsoft, Redmond, WA, USA) was used to calculate 95% confidence intervals (CIs).

All statistical analyses were conducted in Genstat (22nd Edition, VSN International, 2022). For modelling purposes, age was categorised as <2 years, 2–5 years, 5–8 years, and >8 years old. The possible effects of age in years (<2, 2–5, 5–8 or >8), sex (male or female), neuter status (entire or neutered), health status (healthy or sick), and geographic location (NSW, VIC, NT, SA, TAS) on FIV status according to results from blood testing were assessed using logistic regression with an underlying binomial distribution. Univariable analyses were conducted, and any variables with a *p* < 0.25 were considered for inclusion in the multivariable model. A backwards, stepwise approach was used to obtain a final multivariable model in which all variables were significant. In the multivariable model, a *p* value < 0.05 was considered significant.

## 3. Results

### 3.1. Study Population

A total of 382 cats of unknown FIV status were recruited. Cats ranged in age from 0.5 months to 18 years (mean age 5.8 ± 4.3 years). Cats comprised 71 (19%) entire males, 163 (43%) castrated males, 44 (12%) entire females, and 104 (27%) spayed females.

### 3.2. Anigen Rapid^®^ FIV PoC Testing Using Blood

In total, 57/382 (14.9%) cats were FIV-positive with blood testing. There was 100% test agreement between testers. The 57 FIV-positive cats comprised 46/234 (19.6%) males and 11/148 (7.4%) females. The mean age of FIV-positive cats was 6.8 ± 3.4 years.

### 3.3. Anigen Rapid^®^ FIV PoC Testing Using Saliva

In total, 48/382 (12.5%) cats were FIV-positive with saliva testing. There was 100% test agreement between testers.

All 48 cats FIV-positive by saliva were also FIV-positive by blood (i.e., 48 true positives), and no cats FIV-positive by saliva were not FIV-positive by blood (i.e., 0 false positives). Of the 48 saliva FIV-positive results, 43 were strongly positive, 3 were faintly positive, and 2 were very faintly positive. Saliva testing produced 325 true negative FIV results and 9 false negative FIV results. Of the 9 false negative saliva results obtained, 4 (44%) were from the same clinic.

Test sensitivity for Anigen Rapid^®^ FIV using saliva was (48/(48 + 9) × 100) = 84.2% (95% CI 80.6 to 87.9).

Test specificity for Anigen Rapid^®^ FIV using saliva was [(325/(0 + 325) × 100] = 100%.

### 3.4. Summary of FIV Results, Sex, and Health Status

Table 1 summarises the demographic findings for age, sex, and health status, along with blood and saliva FIV results by location (state/territory).

### 3.5. Risk Factors for FIV Infection (Univariable Analysis)

Significant associations between all variables and FIV status were found, except for neuter status and age by numeric value (i.e., geographic location, age category, sex, and health status were all significant; Table 2).

Male cats were more likely to be FIV-positive than female cats (*p* = 0.002). Neuter status did not affect the proportion of FIV-positive cats (*p* = 0.79).

Cats aged between 5 and 8 years were at the greatest likelihood of being infected (23.6% positive), and 5 times (95% CI 1.7–16.7) more likely to be infected than those less than 2 years old. Cats aged between 2 and 5 years and over 8 years were also at higher risk of being positive than cats less than 2 years old (*p* = 0.039).

**Figure 2:**
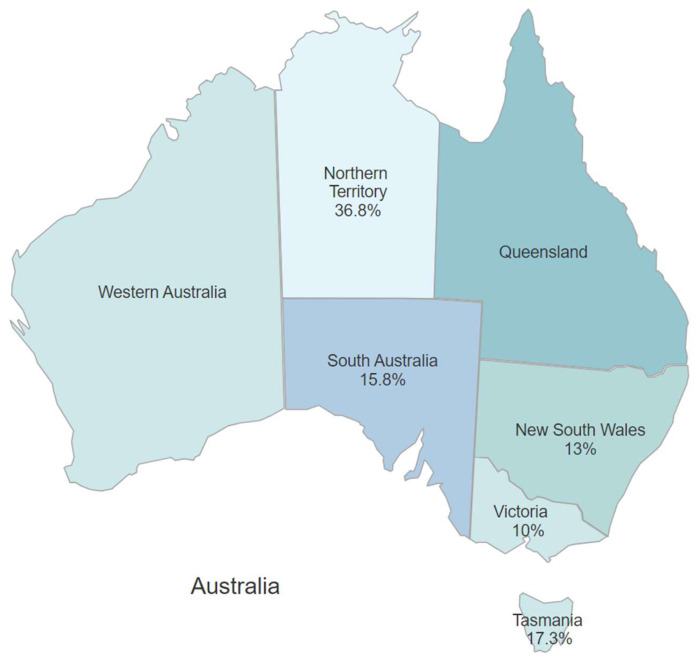
Map of Australia showing the prevalence of FIV-positive cats using blood by state or territory. With univariable analysis, Northern Territory had the highest prevalence of FIV-positive cats. Sampling was not performed in Western Australia or Queensland.

Cats in Victoria were the least likely to be infected (10%), while cats in the Northern Territory were more likely to be positive than cats in other geographical locations (*p* = 0.045). The odds of Northern Territory cats being FIV-positive were 5.25 times (95% CI 1.79–15.4) higher than cats in Victoria, where cats were at the lowest geographical risk (Figure 2).

### 3.6. Risk Factors for FIV Infection (Multivariable Analysis)

Table 3 shows results from the final multivariable model, with sex and health status remaining significant. Male cats had the highest prevalence of infection with 3.1 times (95% CI 1.5–6.19) the risk of being FIV-positive compared to female cats (*p* = 0.001). Sick cats were 2.45 times (95% CI 1.4–4.4) more likely to be FIV-positive than healthy cats (*p* = 0.004). There was no significant interaction between sex and health status (*p* = 0.85).

Geographic location and age by quartile did not retain significance in the multivariable model (*p* = 0.30 and *p* = 0.12, respectively) due to the strength of sex and health status within the model.

## 4. Discussion

This study primarily sought to determine the accuracy of Anigen Rapid^®^ FIV PoC testing using saliva instead of blood as the diagnostic specimen. Although using saliva for Anigen Rapid^®^ FIV testing is ‘off-label’ in Australia (i.e., not a label claim made by the manufacturer or endorsed by the Australian Pesticides and Veterinary Medicines Authority), we believe 84.2% sensitivity and 100% specificity is high enough to recommend its application in healthy animals, particularly in any shelter where high volume screening for FIV infection might be undertaken, and in cats prior to annual FIV re-vaccination. The sole FIV vaccine available in Australia (Fel-O-Vax FIV^®^, manufactured in IA, USA; distributed by Boehringer Ingelheim Animal Health in Australia; also available in Tokyo, Japan) does not provide 100% protection, and therefore experts have recommended annual FIV testing to identify ‘vaccine breakthroughs’ and prevent unnecessary re-vaccination [14,15,16].

The purpose of this study was to provide clinicians an evidence-based methodology for FIV testing that does not require a blood test but instead a sample of saliva that can quickly and easily be used to determine whether a cat is infected with FIV or not. The sample of saliva is therefore key to the outcomes sought in many clinical scenarios. Correct diagnostic and therapeutic decisions rely on the accuracy of test results and the accuracy of test results is dependent on the quality of the sample.

In human medicine, there are multiple different approaches to obtaining saliva for clinical applications, including intraoral duct cannulation, gentle suction, or using custom-made devices for the selective sampling of specific glands as well as whole saliva sampling [17]. Despite the high sensitivity and specificity of saliva FIV testing in the current study, there remains no standardised methodology for saliva collection for cats. Many researchers report the use of sterile cotton swabs, buffered saline, and centrifugation [9,10,12,18,19]. In a side quest for simplicity and practicality, a previous group piloted a revised ‘direct’ technique in a small subset of cats (*n* = 14) using standard, readily available, non-sterile cotton-tips and an adjustment to the manufacturer’s instructions for Anigen Rapid^®^ FIV kits. The preliminary results were promising, with 100% sensitivity obtained, including one FIV-infected cat that was blood-negative/saliva-positive with Anigen Rapid^®^ FIV [12].

In this study, the same ‘direct’ collection technique was employed by using a conventional cotton-tip swab onto the oral mucosa and then applying it directly onto the test well. This technique has the advantage of being low cost, easy (technically easier than blood collection), relatively safe, and suitable for most cats. Shelter cats, who were predominantly strays and frequently quite fractious, tolerated the saliva collection well, especially as no clipping of fur or forced restraint was required (as is necessary with blood collection). However, while many cats were amenable to saliva collection, not all cats were accepting of the process. Occasionally cats would attempt to chew the cotton bud, but this was uncommon. Additionally, it was not always possible to visually determine if an adequate sample volume of saliva had been collected. Saliva collection was found to be inconsistent, with some swabs appearing very dry whereas others were obviously moist and saliva laden. Some swabs had slight blood contamination, presumably due to gingivostomatitis and/or dental disease. Unfortunately, the possible impact of sample quality on saliva results could not be assessed (particularly in relation to the nine false negative saliva FIV results), since a description of the sample quality was infrequently provided by recruiting veterinarians. This remains an area of speculation in terms of the impact on results and potentially an area for future work. In this study, the sample collection technique was not monitored or assessed, rather, relied on adherence to the video SOP provided to clinicians. In practice, a clinician may be uncertain as to the quality of the sample obtained, particularly in a conscious, uncooperative cat and where the swab appears dry. At this time, if saliva FIV testing is adopted, we would advise re-sampling if the cotton tip does not appear moist or saliva is not grossly evident following swabbing. Alternatively, the use of specialised flock-tipped swabs could be employed since they have no internal core to trap the sample, allowing rapid adsorption and fast and complete specimen elution. Although contrary to the intent of using accessible and low-cost consumables, swabs designed for saliva testing offer a further interesting research opportunity and the possibility of improved test sensitivity.

Salivary secretion is affected by endogenous, mechanical, gustatory, and olfactory stimuli with flow and composition being regulated by the autonomic nervous system (ANS) [20]. Sedative drugs affecting the ANS, therefore, can impact on salivary volume and composition, and may in turn affect the accuracy of saliva testing. Drugs such as atropine and glycopyrrolate are known to decrease saliva production as could stress due to a reduction in blood flow to the salivary glands. However, cats are also known to salivate in both stressful and relaxing situations, and sedation could actually enhance saliva collection through the resulting patient compliance. In this study, cats were not routinely sedated for sampling, although some samples may have been obtained under sedation administered for an unrelated reason at the discretion of the consulting veterinarian (e.g., treatment of a cat bite abscess, dental procedure, etc.).

One collection site (Clinic 12, Table 1) had a substantially lower sensitivity rate with FIV saliva testing than the other sites. At this clinic in Tasmania, 30% of the cats sampled were FIV-positive on blood sampling whereas only 10% were FIV-positive with saliva. The six FIV-positive cats were predominantly healthy. The two true positive cats with saliva (positive on blood and saliva) were both presented for oral conditions-one for a dental procedure and one for treatment of stomatitis. Cats with oral cavity disease may have increased saliva production and/or blood contamination when sampling that may enhance FIV testing with saliva. The four false positive cats presented either as healthy or with conditions unrelated to the mouth. It is unknown whether any of these six cats were sedated prior to sampling. Another possible explanation for the false negative saliva results is the immunoglobulin dilution effect that occurs in saliva. In people, the concentration of total IgG in whole saliva is approximately 800–1000 times less than in plasma [8,21], while in healthy cats whole saliva contains 190 to 340 times less IgG than serum [22].

A secondary aim of the study was to investigate FIV prevalence in various Australian states and territories, including Tasmania and the Northern Territory for which prevalence data were not previously available. It has been reported that Western Australia has a significantly higher FIV seroprevalence compared with elsewhere in the country [23,24,25]. Here, it was found that cats in the Northern Territory were at higher risk of being infected in the univariable analysis, but this apparent high prevalence did not translate across to significance in the multivariable model, most likely due to small sample numbers and high standard error. It is a common occurrence that variables that are significant at a univariable level do not retain significance in the multivariable model. In this case, the effects of sex and health status were so strong in the multivariable model that the lesser effect of location (*p* = 0.045 at univariable level) became insignificant and was therefore not included in the final model.

The ownership status (stray or owned) was not assessed in this study and may be a confounding variable in some geographic locations. We suspect the low FIV prevalence found in Victoria with univariable analysis was due to a high proportion of young shelter cats being tested.

Reported risk factors for FIV infection include sex (entire males) and age (older than three years) [25,26,27,28,29,30,31,32,33,34,35,36,37,38,39]. This study corroborates that male cats are at higher risk of infection in Australia [25,33,40]. Interestingly, while age has previously been found to be a risk factor in Australia, this was not supported in the current study after adjusting for other terms in the multivariable model, despite 5–8-year-old cats having a higher risk of infection than other age groups with univariable analysis. Logically, older cats have more chance to be infected because they have more opportunity to fight; however, both anecdotal evidence (in high-volume shelters) and evidence in other studies have found that FIV-infected cats may experience a normal length of life with appropriate husbandry and disease management [41,42,43,44]. Thus, a positive result should not be a reason for euthanasia, and in fact, any decisions on the outcome for positive animals should not be made without confirmatory testing being performed, as described in the AAFP retroviral guidelines and the Australia and New Zealand FIV guidelines [1,16].

It was variously reported in the literature that sick cats have a higher prevalence of FIV positivity. The determination of what constitutes an animal to be ‘sick’ is subjective, and the variability in findings across different studies could simply be indicative of the rigour in which sick versus healthy definitions are applied [25,26,28,31,33,34,40,45]. The current study found a significant association between health status and FIV status, with ‘sick cats’ more likely to be FIV-positive than healthycats. Sick cats encompass a range of presentations not limited to those that might be associated for FIV. Although this is not evidence of direct causation, this finding does support a broad collection of other studies that have reported negative health consequences from FIV infection [25,26,28,31,34,40,45]. Consequently, testing should be a priority in cats with recurrent infections, unexplained neutropenia, anaemia, or lymphoma.

As per the AAFP retroviral guidelines and the Australia and New Zealand FIV guidelines, PoC tests are the recommended first diagnostic step, but since no test has 100% sensitivity and specificity, a second confirmatory test should be undertaken if the first result is positive [1,16]. Negative FIV test results are generally reliable, particularly in low-risk individuals, e.g., healthy females in a stable household. However, the false negative results obtained here may be of concern to some. It should be stated, however, that in typical cat populations with a seroprevalence of 1–5%, false positives can more readily occur [46]. Since a false positive FIV result could lead to unnecessary segregation or even euthanasia, a false negative FIV result is, in fact, less risk to an individual’s life but a greater herd-health risk. Clinicians are referred again to the AAFP retroviral guidelines and Australia and New Zealand FIV guidelines, specifically to the use of an additional alternate brand of PoC test or PCR testing in specific circumstances [1,16].

Although blood remains the sample of choice for testing with Anigen Rapid^®^ FIV and is recommended to be used to determine FIV status in any situation where blood is already being collected, we believe findings from this study should give clinicians considerable confidence to use saliva instead of blood for first line FIV diagnosis in certain instances, particularly FIV screening in shelters. This may encourage some shelters that are moving away from routine FIV testing for logistical reasons to reconsider due to the ease of diagnosis since, even though many FIV-infected cats can live asymptomatically for many years, they must be managed correctly if they are to be co-housed with FIV-uninfected cats [1,16]. If saliva is not grossly visible on the cotton tip, attempts should be made to recollect further sample. Clinicians can encourage salivation with impunity, for example, by petting a co-operative patient or using tempting food, since the stimulation of saliva and the retention of analytes are not factors in the assessment of viruses. When there is significant concern for a false negative saliva FIV result (e.g., in a male cat with a history of fighting), a follow-up blood sample should be considered for FIV testing.

## 5. Conclusions

Using a simple, direct, and fast method to test saliva, the sensitivity (84.2%) and specificity (100%) of Anigen Rapid^®^ FIV was high enough to recommend its routine application in healthy animals, particularly in any shelter where high volume screening for FIV infection might be undertaken. Furthermore, male cats and sick cats were identified as the dominant risk factors for FIV infection in Australia, and efforts to ascertain the FIV status of these cats should be redoubled by clinicians.

## Figures and Tables

**Table 1:** FIV and demographic results by location (state/territory) in Australia. SD = standard deviation.

Location	Group	Mean Age ± SD (Years)	Sex (Female/Male)	Healthy (%)	FIV Result–BLOOD (%)	FIV Result– SALIVA (%)
Victoria (*n* = 150)	Shelter 1	3.2 ± 2.7	58:92	141/150 (94%)	15/150 (10%)	13/150 (9%)
New South Wales (*n* = 23)	Teaching Hospital	9.5 ± 6.1	10:13	16/23 (70%)	3/23 (13%)	3/23 (13%)
Northern Territory (*n* = 19)	Clinic 1 (*n* = 9)	9.1 ± 3.9	5:4	0/9 (100%)	1/9 (11%)	1/9 (11%)
	Clinic 2 (*n* = 6)	4.7 ± 2.1	1:5	5/6 (83%)	4/6 (67%)	4/6 (67%)
	Shelter 2 (*n* = 4)	7 ± 2.4	1:3	1/4 (25%)	2/4 (50%)	2/4 (50%)
SUBTOTAL		7.5 ± 3.6	7:12	6/19 (32%)	7/19 (37%)	7/19 (37%)
South Australia (*n* = 57)	Clinic 3 (*n* = 6)	8.3 ± 4.2	3:3	5/6 (83%)	1/6 (17%)	0/6 (0%)
	Clinic 4 (*n* = 10)	4.6 ± 5.2	3:7	7/10(70%)	2/10 (20%)	2/10 (20%)
	Clinic 5 (*n* = 5)	3.6 ± 1.6	0:5	5/5 (100%)	0/5 (0%)	0/5 (0%)
	Clinic 6 (*n* = 9)	6.6 ± 4.6	2:7	9/9 (100%)	0/9 (0%)	0/9 (0%)
	Clinic 7 (*n* = 8)	6.4 ± 3.2	2:6	7/8 (87%)	1/8 (13%)	1/8 (13%)
	Clinic 8 (*n* = 4)	8 ± 3.3	1:3	4/4 (100%)	0/4 (0%)	0/4 (0%)
	Clinic 9 (*n* = 7)	6.7 ± 3.8	4:3	3/7 (43%)	2/7 (29%)	2/7 (29%)
	Clinic 10 (*n* = 8)	7.1 ± 4.5	1:7	5/8 (62%)	3/8 (38%)	3/8 (38%)
SUBTOTAL		6.5 ± 4.1	16:41	45/57 (79%)	9/57 (16%)	8/57 (14%)
Tasmania (*n* = 133)	Clinic 11 (*n* = 30) Clinic 12 (*n* = 20)	7.5 ± 4.2 7.5 ± 3.8	12:18 9:11	14/30 (47%) 11/20 (55%)	6/30 (20%) 6/20 (30%)	6/30 (20%) 2/20 (10%)
	Clinic 13 (*n* = 8)	6.7 ± 5.4	3:5	7/8 (87%)	2/8 (25%)	2/8 (25%)
	Clinic 14 (*n* = 7)	9.7 ± 5.3	3:4	2/7(28%)	1/7 (14%)	1/7 (14%)
	Clinic 15 (*n* = 17)	7.5 ± 3.9	11:6	14/17(82%)	1/17 (6%)	0/17 (0%)
	Clinic 16 (*n* = 15)	9.0 ± 4.4	6:9	9/15 (65%)	2/15 (13%)	1/15 (7%)
	Clinic 17 (*n* = 17)	6.7 ± 4.1	7:10	8/17 (47%)	3/17 (18%)	3/17 (18%)
	Clinic 18 (*n* = 2)	8.8 ± 0.2	0:2	1/2 (50%)	0/2 (0%)	0/2 (0%)
	Clinic 19 (*n* = 12)	8.0 ± 3.8	4:8	6/12 (50%)	2/12 (17%)	2/12 (17%)
	Clinic 20 (*n* = 5)	5.0 ± 3.4	1:4	3/5 (60%)	0/5 (0%)	0/5 (0%)
SUBTOTAL		7.6 ± 4.1	56:77	75/133 (56%)	23/133 (17%)	17/133 (13%)
TOTAL (*n* = 382)		5.8 ± 4.3	147:235	283/382 (74%)	57/382	48/382

**Table 2:** Results from univariable logistic regression modelling of variables possibly affecting FIV status. S.E. = standard error; ref = reference.

		FIV Prevalence (%)	S.E.	Odds Ratio	95% CI	*p* Value
Geographic location	Victoria	10.0	2.4	ref		0.045
	New South Wales	13.0	7.02	1.35	0.36–5.1	
	Northern Territory	36.8	11.1	5.25	1.79–15.4	
	South Australia	15.8	4.8	1.68	0.69–4.1	
	Tasmania	17.3	3.3	1.88	0.94–3.8	
Age category (years)	<2	5.5	2.7	ref		0.039
	2–5	15.7	3.5	3.2	1.04–9.98	
	5–8	23.6	5	5.0	1.70–16.72	
	>8	16.0	3.4	3.5	1.07–10.03	
Sex	Female Male	7.4 19.6	2.2 2.6	ref 3.05	1.52–6.09	0.002
Neuter status	Entire Neutered	15.6 14.6	3.4 2.2	ref 0.92	0.50–1.69	0.79
Health	Healthy	11.6	1.7	ref		0.003
	Sick	24.4	3.9	2.42	1.42–4.13	

**Table 3:** Results from multivariable logistic regression modelling of variables affecting FIV status. S.E. = standard error; ref = reference.

		FIV Prevalence (%)	S.E.	Odds Ratio	95% CI	*p* Value
Sex	Female Male	7.5 19.6	2.2 2.5	ref 3.08	1.53–6.19	0.001
Health Status	Healthy	11.7	1.85	ref		0.004
	Sick	24.0	4.20	2.45	1.35–4.44	

## Data Availability

All the data presented in this paper are available on request.
